# Serum lactate dehydrogenase is associated with the presence and extent of preoperative peritumoral edema in melanoma brain metastases

**DOI:** 10.1007/s11060-026-05681-6

**Published:** 2026-07-06

**Authors:** Alim Emre Basaran, Luca Fahsold, Martin Vychopen, Alonso Barrantes-Freer, Wolf C. Müller, Jan C. Simon, Mirjana Ziemer, Erdem Güresir, Johannes Wach

**Affiliations:** 1 Department of Neurosurgery, University Hospital Leipzig, University of Leipzig, Liebigstr. 20, 04103 Leipzig, Germany; 2 Comprehensive Cancer Center Central Germany, Partner Site Leipzig, 04103 Leipzig, Germany; 3 Paul-Flechsig-Institute of Neuropathology, University Hospital Leipzig, 04103 Leipzig, Germany; 4 Department of Dermatology, Venerology and Allergology, University of Leipzig Medical Center, Leipzig, Germany

**Keywords:** Melanoma brain metastases, Peritumoral brain edema, Lactate dehydrogenase, Biomarker, Preoperative risk stratification

## Abstract

**Background:**

Peritumoral brain edema (PTBE) is a major contributor to neurological morbidity in patients with melanoma brain metastases (BM). While serum lactate dehydrogenase (LDH) is an established systemic biomarker in metastatic melanoma, its relationship with local radiological characteristics such as PTBE remains insufficiently understood. This study aimed to investigate the association between serum LDH levels and PTBE, as well as its relationship with clinical and tumor-related parameters.

**Methods:**

We performed a retrospective analysis of 56 consecutive patients who underwent surgical resection for melanoma BM between 2012 and 2024. Preoperative MRI was used to assess the presence and volumetric extent of PTBE. Clinical, radiological, and laboratory parameters, including serum LDH levels, MIB-1 proliferation index, tumor volume and preoperative seizures were analyzed. Associations were evaluated using univariate analyses and Spearman rank correlation. Receiver operating characteristic (ROC) curve analyses were performed to assess the discriminative ability of the variables.

**Results:**

Preoperative PTBE was present in 71.4% of patients. Serum LDH levels were significantly higher in patients with PTBE (*p* < 0.001) and demonstrated a strong positive correlation with edema volume (*r* = 0.822, *p* < 0.001). In addition, LDH levels were significantly correlated with the MIB-1 proliferation index (*r* = 0.601, *p* < 0.001) and tumor volume (*r* = 0.584, *p* < 0.001) and were significantly elevated in patients presenting with preoperative seizures (*p* < 0.001). ROC analysis demonstrated that LDH had the highest discriminative performance (AUC 0.802, *p* < 0.001), with an optimal cut-off value of 179.4 U/L.

**Conclusions:**

In this exploratory study, serum LDH was associated with both the presence and extent of PTBE and may serve as a surrogate of tumor-related biological activity. These hypothesis-generating findings warrant prospective validation before clinical implications can be drawn.

**Graphical Abstract:**

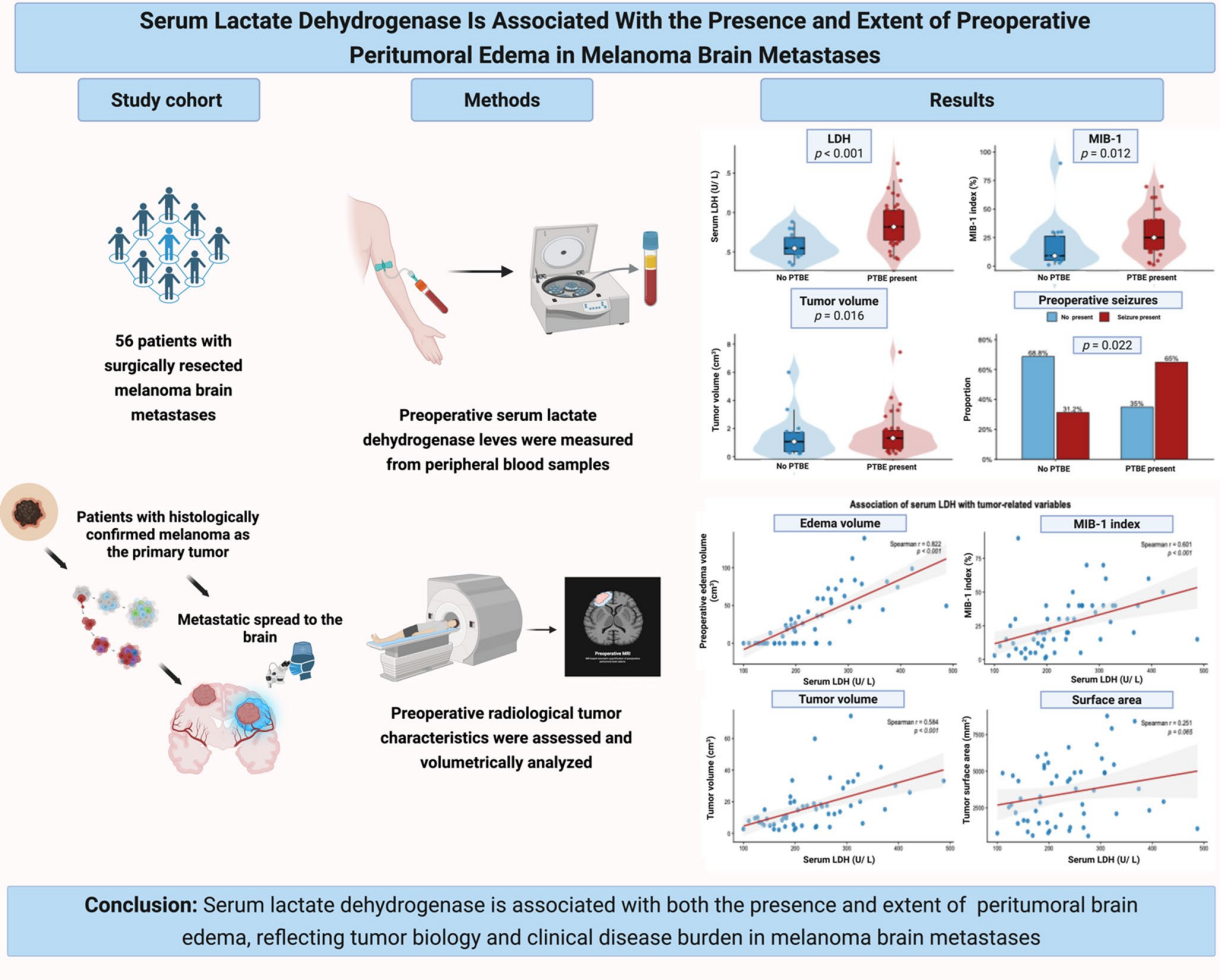

## Introduction

Brain metastases (BM) are among the most common intracranial tumors in adults and occur in up to 40% of patients with advanced malignancies. The management of patient with melanoma BM requires individualized preoperative risk assessment, as the extent of PTBE substantially influences neurological morbidity, perioperative planning and the need for corticosteroid therapy [1, 2]. Identifying simple, routinely available preoperative biomarkers that reflect the likelihood and severity of PTBE could therefore represent a meaningful step toward more individualized perioperative care. Melanoma has a particularly high propensity to metastasize to the brain, with approximately 40–60% of patients develop BM during the course of disease [3–5]. Despite considerable progress in systemic therapies for melanoma, BMs remain a major cause of neurological deficits, reduced quality of life (QoL) and mortality in this patient population [6–8]. In addition to the direct space-occupying effect of the tumor, peritumoral brain edema (PTBE) plays a crucial role in the development of neurological symptoms such as headache, focal neurological deficits and seizures. PTBE in BM is primarily considered as a vasogenic edema resulting from disruption of the blood-brain barrier (BBB) [9, 10]. Tumor-associated mechanisms, including increased vascular permeability, activation of inflammatory signal pathways and the release of angiogenic factors particularly vascular endothelial growth factor (VEGF) all contribute to the development of PTBE [11]. Previous studies have demonstrated that the extent of PTBE is influenced by tumor-related biological characteristics such as tumor volume, proliferative activity and angiogenic potential [12, 13]. Serum lactate dehydrogenase (LDH) is an established biomarker in patients with metastatic melanoma. Elevated serum LDH levels have been associated with increased tumor burden, aggressive disease behavior and poor prognosis [14, 15]. Although LDH is well established as a systemic prognostic marker, its relationship with local radiological characteristics of BM, particularly the development and extent of PTBE remains poorly understood.

The aim of the present study was therefore to investigate whether preoperative serum LDH levels a routinely available laboratory parameter are associated with the presence and volumetric extent of PTBE in patients with melanoma BM and to examine its relationship with clinical and tumor-related characteristics. By identifying LDH as a potential surrogate of PTBE burden, we aimed to explore its utility as simple preoperative biomarker to support individualized perioperative risk stratification in this patient population.

## Materials and methods

### Study population

**Fig. 1:**
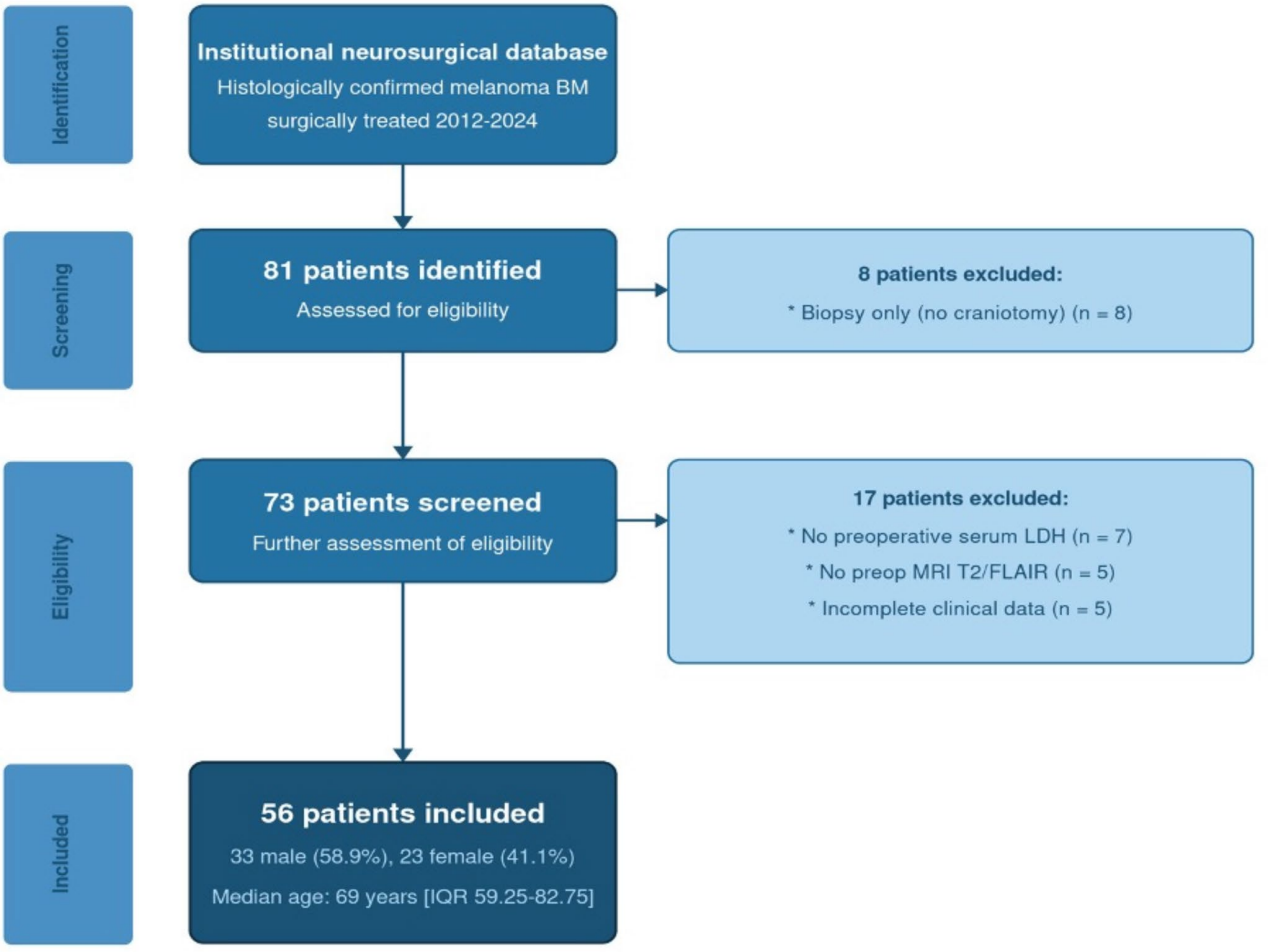
A total of 81 patients with histologically confirmed melanoma brain metastases who underwent surgical resection between 2012 and 2024 were screened. Eight patients were excluded due to biopsy-only procedures without craniotomy, leaving 73 patients assessed for eligibility. Of these, 17 were further excluded due to absence of preoperative serum LDH (*n* = 7), unavailability of preoperative MRI with T2/FLAIR sequences (*n* = 5), or incomplete clinical data (*n* = 5). Ultimately, 56 patients were included in the final analysis. BM, brain metastases; FLAIR, fluid-attenuated inversion recovery; LDH, lactate dehydrogenase; MRI, magnetic resonance imaging

This retrospective study included consecutive patients who underwent surgical resection of melanoma BM at the Department of Neurosurgery, University Medical Center Leipzig, between 2012 and 2024. All patients were managed according to standardized institutional protocols for perioperative neurosurgical care, including preoperative corticosteroid administration for symptomatic PTBE, seizure prophylaxis in patients with a documented preoperative seizure history, routine preoperative laboratory workup including serum LDH measurement and standardized postoperative neurological monitoring with corticosteroid tapering. Eligible cases were identified through our institutional neurosurgical database using standardized ICD-coding, with subsequent extraction of clinical, laboratory and imaging data from the electronic patient record system. The diagnosis of brain metastasis was confirmed histopathologically following surgical resection in all cases. The underlying primary diagnosis of melanoma had been established histopathologically prior to surgery in all patients either through prior biopsy of the primary cutaneous or mucosal lesion, biopsy of extracranial metastatic disease or in cases where brain metastases represented the initial manifestation of metastatic disease, through histopathologically analysis of the surgically resected brain lesion. The patient selection process is illustrated in Fig. 1.

### Radiological assessment

Preoperative magnetic resonance imaging (MRI) was reviewed in all patients. Tumor imaging characteristics including tumor location, tumor volume, surface area, and roundness were assessed on contrast-enhanced T1-weighted gadolinium (Gd)-enhanced MRI. Image segmentation and quantitative analysis of these morphometric tumor features were performed using the open-source software platform 3D Slicer (version 5.2.1, Surgical Planning Laboratory, Harvard University, USA) [16]. Tumor segmentation was conducted using a semi-automated workflow to derive volumetric and geometric tumor parameters. Roundness was calculated as the ratio of the tumor volume to the volume of a sphere with a radius equal to the maximum distance from the centroid to the tumor surface. This dimensionless parameter ranges from 0 to 1, with values approaching indicating a spherical shape and lower values reflecting increasing irregularity [17]. All segmentations were performed by a single experienced rater. Secondary review was performed by a senior neurosurgeon (J.W.). Any disagreement was resolved by a third senior neurosurgeon (M.V.)

The presence of PTBE was assessed on preoperative MRI using fluid-attenuated inversion recovery (FLAIR) where available. In cases where FLAIR sequences were not included in the preoperative imaging protocol, T2-weighted sequences were used as an alternative. PTBE was defined as hyperintense signal surrounding the lesion consistent with vasogenic edema. In addition, PTBE volumes were quantified using a semi-automated segmentation approach with the SmartBrush tool implemented in the BrainLAB software platform (BrainLAB^®^, Munich, Germany).

### Clinical variables and laboratory parameters

Clinical data were obtained from institutional medical records. The collected variables included patient age at surgery, preoperative seizure presentation, Karnofsky Performance Status (KPS) and the melanoma-specific Graded Prognostic Assessment (GPA) index [18]. Extracranial metastatic disease was defined as the presence of one or more extracranial metastatic manifestations documented at the time of surgery. Preoperative seizure occurrence was defined as any clinically documented seizure event prior to surgical treatment, including both witnessed seizures and seizure events reported in the clinical history. Witnessed seizures and patient-reported events were not recorded separately in the present retrospective analysis. Preoperative LDH levels were obtained from routine laboratory testing performed prior to surgery as part of the standard preoperative diagnostic workup. LDH values were recorded as continuous variables. Measurements were performed using a standardized enzymatic ultraviolet (UV) assay according to the International Federation of Clinical Chemistry (IFCC) reference procedure, based on the rate of NADH formation at 340 nm [19].

### Statistical analyses

Statistical analyses were performed using SPSS (Version 29; IBM, Armonk, NY, USA). Continuous variables were expressed as median with interquartile range [IQR]. Normality of continuous variables was assessed using the Shapiro-Wilk test. As all variables were non-normally distributed, non-parametric tests were applied throughout. Comparisons between groups were performed using the Mann-Whitney U test for continuous variables and the Chi-square test or Fisher´s exact test for categorical variables. Spearman rank correlation was used to assess associations between continuous variables. Scatterplots were generated prior to correlation analyses to visually inspect relationship and identify potential outliers.

Receiver operating characteristics (ROC) curve analysis was performed to evaluate the discriminatory ability of continuous variables for predicting the presence of preoperative PTBE. The optimal cut-off value for each variable was determined using the Youden index. Exploratory pairwise comparisons of areas under curve (AUC) were additionally performed using approximate Z-tests based on estimated standards errors derived from confidence intervals (CI). ROC curves were generated in R using the *ggplot2* and *pROC* packages (R Foundation for statistical Computing, Vienna, Austria). Additionally, an exploratory binary logistic regression analysis was performed with PTBE presence as the dependent variable. All variables reaching statistical significance in the univariate analyses were entered in the multivariable analyses. Given the limited sample size and the number of events per variable below the recommended threshold of 10, this analysis is strictly exploratory and should be interpreted as hypothesis-generating only [20]. A *p*-value < 0.05 was considered statistically significant.

## Results

### Patient characteristics

A total of 56 patients who underwent surgical resection for melanoma BM were included in the study. The cohort consisted of 33 male (58.9%) and 23 female (41.1%) patients. The median age at the time of surgery was 69 years [59.25–82.75].

Regarding tumor localization, the frontal lobe was the most frequent site (39.3%), followed by the parietal lobe (25.0%), cerebellum (16.1%), temporal lobe (10.7%) and occipital lobe (7.1%). One lesion (1.8%) was located in the brainstem.

The median preoperative serum LDH level was 213.9 U/L [175.5–284.1]. BRAF-V600 mutation was present in 11 patients (19.6%). Multiple intracranial metastases were observed in 25 patients (44.6%), while 31 patients (55.4%) presented with a solitary lesion. In addition, extracranial metastatic disease was present in 41 patients (73.2%). The remaining 15 patients (26.8%) presented with brain metastases as the sole site of metastatic spread, with a histologically confirmed primary tumor but no evidence of additional systemic metastases at the time of surgery. Regarding the distribution of extracranial metastatic manifestations, lymph node metastases were present in 48 patients (85.7%) and osseous metastases in 13 patients (23.2%). Of the 56 patients included, 23 (41.1%) were previously untreated at the time of surgery, defined as having received neither prior immunotherapy nor prior systemic chemotherapy for metastatic melanoma. The remaining 33 patients (58.9%) had received prior systemic therapy: 26 patients (46.4%) had received immune checkpoint inhibitor therapy and 9 patients (16.1%) had received prior chemotherapy, with 2 patients having received both modalities. Additionally, 15 patients (26.8%) had undergone prior cranial radiotherapy, including stereotactic or whole-brain radiotherapy. Regarding preoperative corticosteroid use, 30 patients (53.6%) received corticosteroid treatment. Necrosis was observed on preoperative MRI in all patients.

Preoperative PTBE was present in 40 patients (71.4%), whereas 16 patients (28.6%) showed no edema on imaging. The median preoperative edema volume was 23.75 cm³ [0.0–56.85].

The median tumor volume was 13.69 cm^3^ [5.8–20.21], and the median tumor surface area was 31.23 cm² [14.98–49.17]. Morphometric tumor characteristics showed a median roundness of 0.17 [0.15–0.20]. Histopathological assessment demonstrated a median MIB-1 proliferation index of 20% [10.5–40.0]. The median GPA index of the cohort was 2.5 [2.0–3.5]. Additionally, the median preoperative KPS score in the overall cohort was 70.0 [70.0-87.5]. Summarized patient characteristics are provided in Table 1.

**Table 1:** Patient characteristics

Parameter	Value
**Sex**	
Male	33/56 (58.9%)
Female	23/56 (41.1%)
**Age at surgery, median [IQR]**	69 [59.25-82.75]
**Neuroanatomical location**	
Frontal	22/56 (39.3%)
Temporal	6/56 (10.7%)
Parietal	14/56 (25.0%)
Occipital	4/56 (7.1%)
Cerebellar	9/56 (16.1%)
Brainstem	1/56 (1.8%)
**LDH (U/L), median [IQR]**	213.9 [175.5-284.1]
**BRAF mutation status**	
Present	11/56 (19.6%)
Absent	45/56 (80.4%)
**Additional brain metastases**	
Yes	25/56 (44.6%)
No	31/56 (55.4%)
**Extracranial metastatic disease, n (%) ** ^†^	
Yes	41/56 (73.2%)
No	15/56 (26.8%)
^‡^ **Lymph node metastases**	
Yes	48/56 (85.7%)
No	8/56 (14.3%)
**Osseous metastases **	
Yes	13/56 (23.2%)
No	43/56 (76.8%)
**Prior immunotherapy**	
Yes	26/56 (46.4%)
No	30/56 (53.6%)
**Prior chemotherapy**	
Yes	9/56 (16.1%)
No	47/56 (83.9%)
**Prior cranial radiotherapy**	
Yes	15/56 (26.8%)
No	41/56 (73.2%)
**Previously treated**	
Yes	33/56 (58.9%)
No	23/56 (41.1%)
**Preoperative corticosteroid**	
Yes	30/ 56 (53.6%)
No	26/ 56 (46.4%)
**Preoperative Necrosis on MRI**	
Yes	56/56 (100.0%)
No	0/56 (0.0%)
**Preoperative PTBE**	
Present	40/56 (71.4%)
Absent	16/56 (28.6%)
**Preoperative PTBE volume, median [IQR]**	23.75 [0.0-56.85]
**Mib-1 index, median [IQR]**	20.0 [10.5-40.0]
**Tumor volume in cm**^3^, **median [IQR]**	13.69 [5.8–20.21]
**Tumor surface area** (cm^2^), **median [IQR]**	31.23 [14.98-49.17]
**Tumor roundness**	0.17 [0.15-0.2]
**GPA index, median [IQR]**	2.5 [2.0-3.5]
**Preoperative KPS score, median [IQR]**	70.0 [70.0-87.5]

^†^Defined as the presence of one or more extracranial metastatic manifestations documented at the time of surgery. The 15 patients (26.8%) without extracranial metastatic disease presented with brain metastases as the sole site of metastatic spread from a histologically confirmed primary tumor

^‡^Lymph node metastases were assessed based on staging examinations performed during the disease course prior to surgery and were not restricted to findings documented at the time of surgical intervention

### Univariate analyses

**Fig. 2:**
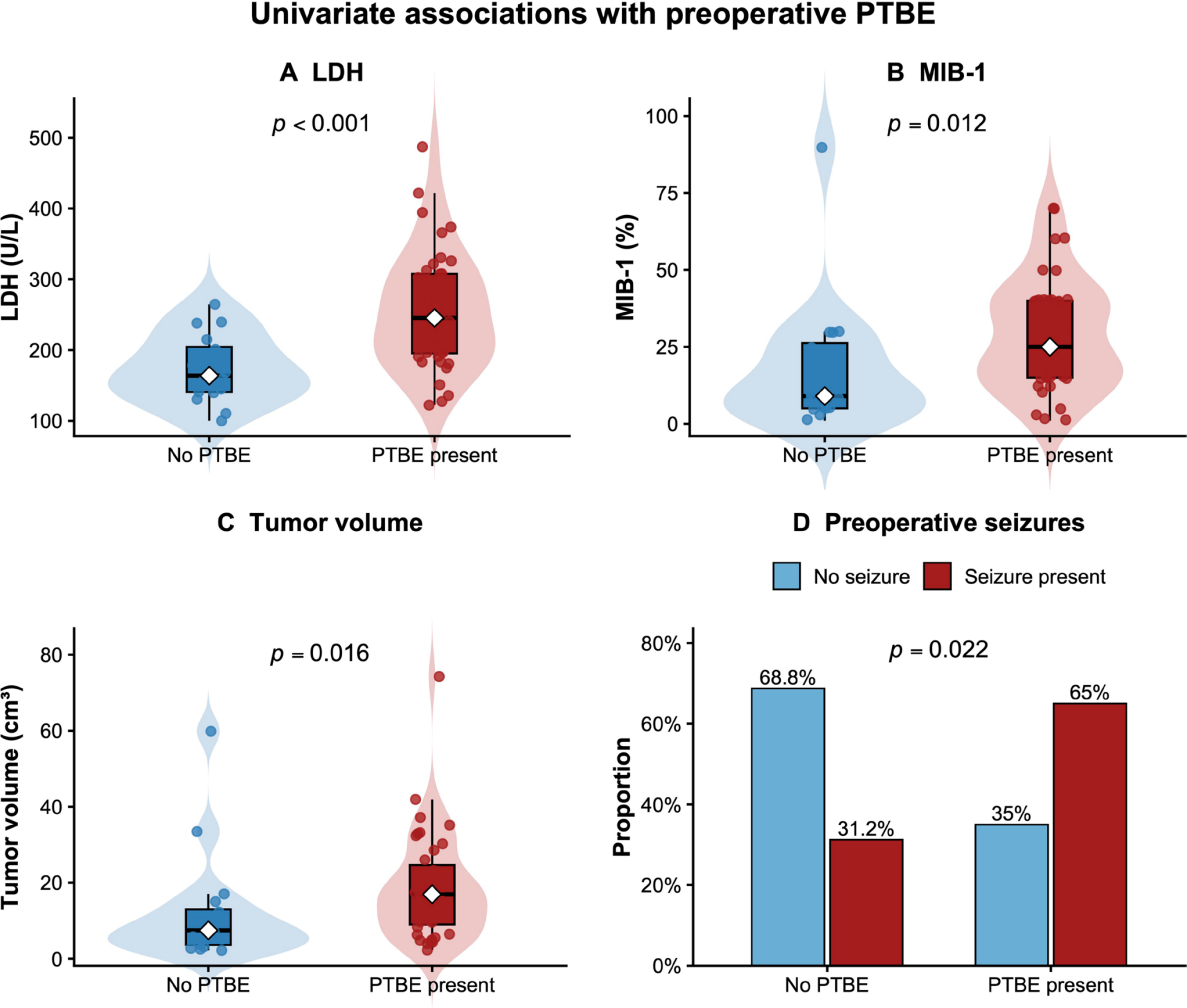
Univariate associations with preoperative PTBE. Violin plots with overlaid boxplots and individual data points illustrate the distribution of continuous variables according to PTBE status (no PTBE vs. PTBE present). Median values are indicated by white diamonds. (**A**) LDH levels were significantly higher in patients with PTBE (*p* < 0.001). (**B**) The MIB-1 proliferation index was significantly elevated in patients with PTBE (*p* = 0.012). (**C**) Tumor volume was significantly associated with the presence of PTBE (*p* = 0.016). (**D**) The proportion of patients with preoperative seizures differed significantly between groups, with a higher frequency observed in patients with PTBE (*p* = 0.022)

Preoperative PTBE was observed in 40 of 56 patients (71.4%), whereas 16 patients (28.6%) showed no PTBE on preoperative MRI. Serum LDH levels were significantly higher in patients with PTBE compared with those without edema (245.4 [192.3-307.8] vs. 163.8 [140.1-211.35] U/L, *p* < 0.001). Beyond the presence of PTBE, serum LDH levels demonstrated a strong positive correlation with the extent of preoperative PTBE, indicating that higher LDH values were associated with larger edema volumes (*r* = 0.822, *p* < 0.001, see Fig. 2**)**. Furthermore, the presence of PTBE was significantly associated with the occurrence of preoperative seizures (*p* = 0.022), suggesting that edema formation is not only a radiological finding but also relates to clinically relevant neurological symptoms.

Higher MIB-1 proliferation indices were significantly associated with the presence of PTBE (28.0 [15.0–40.0]vs. 9.0 [5.0-26.25], *p* = 0.012). Similarly, larger tumor volumes were observed in patients with PTBE (16.93 [8.4–26.1] vs. 7.47 [3.34–14.35] cm^3,^ *p* = 0.016). Age was also significantly associated with PTBE, with younger patients more frequently affected (68.0 [59.0–80.0] vs. 78.5 [69.0–88.0] years, *p* = 0.039). In contrast, sex (*p* = 0.144), BRAF mutation status (*p* = 0.711) and the presence of additional brain metastases (*p* = 0.799) and extracranial metastatic disease (*p* = 0.321) were not significantly associated with PTBE. Furthermore, no significant difference in preoperative KPS score was observed between patients with and without PTBE (*p* = 0.651). These findings suggest that both tumor proliferative activity and tumor burden may be associated with the development of PTBE. Detailed results of the univariate analyses are provided in Table 2, and the significant variables identified (LDH, MIB-1 index, tumor volume, and preoperative seizures) are summarized in Fig. 2.

**Table 2:** Results of univariate analyses

Variable	Total cohort (*n* = 56)	PTBE absent (*n* = 16)	PTBE present (*n* = 40)	*p*-value
Age (years), median [IQR]	69.0 [59.25–82.75]	78.5 [69.0–88.0]	68.0 [59.0–80.0]	*0.039*
LDH (U/L), median [IQR]	213.9 [175.5-284.1]	163.8 [140.1-211.35]	245.4 [192.3-307.8]	*< 0.001*
MIB-1 index (%), median [IQR]	20.0 [10.5–40.0]	9.0 [5.0–26.25]	28.0 [15.0–40.0]	*0.012*
Tumor volume (cm³), median [IQR]	13.69 [5.8–20.21]	7.47 [3.34–14.35]	16.93 [8.4–26.1]	*0.016*
GPA index, median [IQR]	2.5 [2.0–3.5]	3.0 [2.5–3.0]	2.5 [2.0–3.5]	0.716
Male sex, n (%)	33 (58.9%)	7 (43.8%)	26 (65.0%)	0.144
Additional brain metastases, n (%)	25 (44.6%)	4 (25.0%)	21 (52.5%)	0.799
Extracranial metastatic disease, n (%)	41 (73.2%)	10 (62.5%)	31 (77.5%)	0.321
Preoperative seizures, n (%)	31 (55.4%)	5 (31.3%)	26 (65.0%)	*0.022*
BRAF mutation status, n (%)	11 (19.6%)	2 (12.5%)	9 (22.5%)	0.711
Preoperative KPS score, median [IQR]	70.0 [70.0-87.5]	70.0 [70.0-87.5]	70.0 [70.0-87.5]	0.651

### Exploratory multivariable analysis

An exploratory binary logistic regression was performed to assess whether serum LDH remained associated with PTBE after adjustment for other significant univariate predictors. All variables reaching statistical significance in the univariate analyses were entered simultaneously: serum LDH, MIB-1 proliferation index, tumor volume and preoperative seizure occurrence. Given the limited sample size and only 16 patients without PTBE, the number of events per variable was below the recommended threshold of 10 and the results must therefore be interpreted exploratory and susceptible to overfitting [20]. In the multivariable model, MIB-1 proliferation index (OR 12.743, 95% CI: 1.853–87.617, *p* = 0.010) and preoperative seizure occurrence (OR 7.273, 95% CI: 1.136–46.525, *p* = 0.036) retained statistical significance. Serum LDH (OR 1.367, 95% CI: 0.237–7.876, *p* = 0.727) and tumor volume (OR 3.181, 95% CI: 0.642–15.756, *p* = 0.156) did not reach statistical significance. The wide confidence intervals reflect the limited sample size. These findings suggest that the association between LDH and PTBE may be confounded by tumor proliferative activity and should not be interpreted as evidence of an independent effect.

### Association of serum LDH with clinical and tumor-related variables

**Fig. 3:**
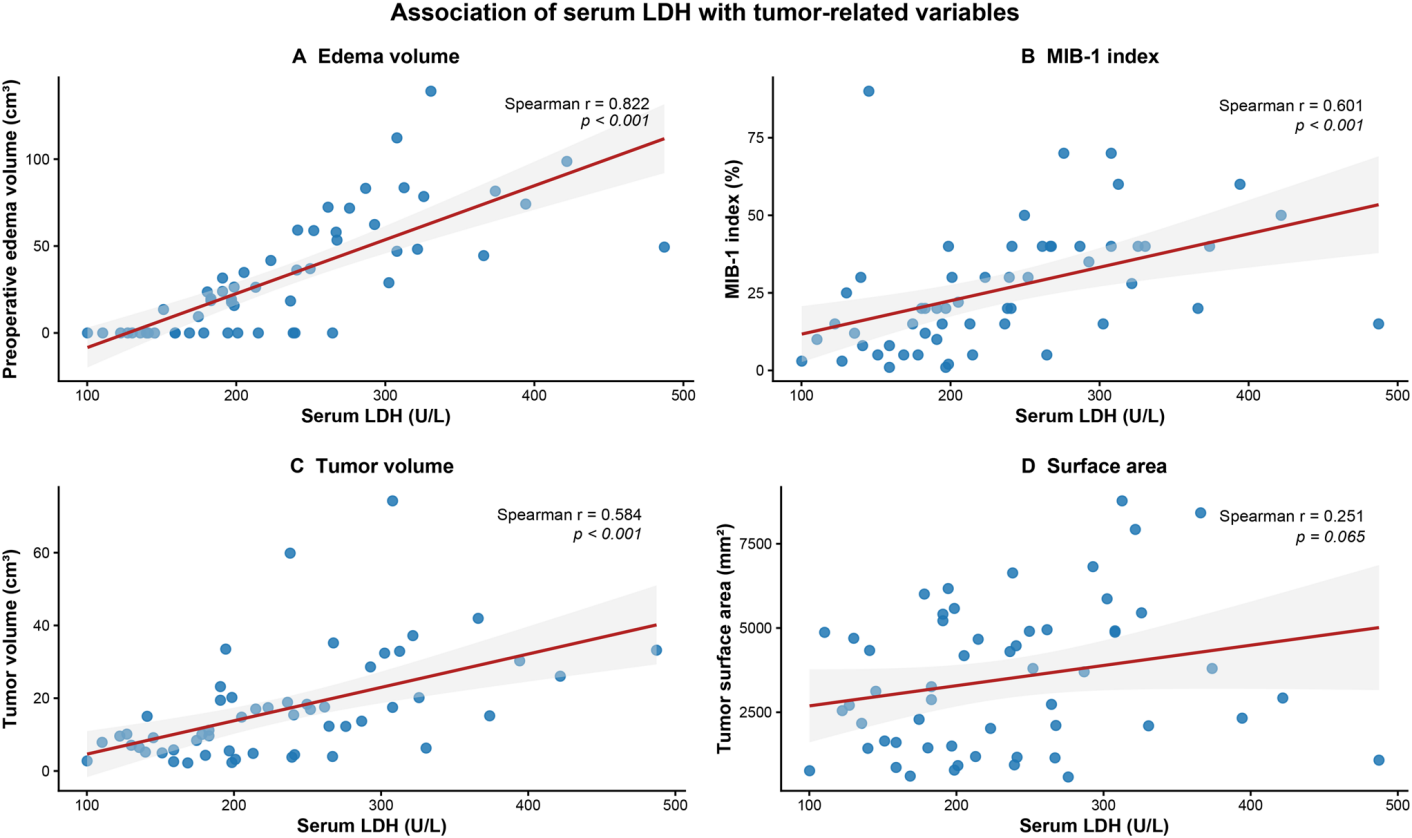
Serum LDH is associated with tumor burden and proliferative activity. Multi-panel scatterplots demonstrating the relationship between preoperative serum LDH levels and tumor-related parameters. (**A**) LDH showed a strong positive correlation with preoperative edema volume (*r* = 0.822, *p* < 0.001) and the (**B**) MIB-1 proliferation index (*r* = 0.601, *p* < 0.001) and a weaker but significant association with (**C**) tumor volume (*r* = 0.584, *p* < 0.001), while no significant correlation was observed with (**D**) tumor surface area (*r* = 0.251, *p* = 0.065). Each point represents an individual patient. Lines indicate a linear regression with 95% confidence intervals

Given the significant association between LDH and PTBE, further analyses were performed to investigate the relationship between serum LDH levels and clinical as well as tumor-related variables. Consistent with the univariate analyses, serum LDH levels demonstrated a strong positive correlation with preoperative edema volume (Spearman *r* = 0.822, *p* < 0.001). In addition, LDH levels were significantly correlated with the MIB-1 proliferation index (*r* = 0.601, *p* < 0.001) and tumor volume (*r* = 0.584, *p* < 0.001), indicating that higher LDH values were associated with increased tumor proliferative activity and tumor burden. No significant correlation was observed between LDH and tumor surface area (*r* = 0.251, *p* = 0.065). These associations are illustrated in Fig. 3. Furthermore, LDH levels were significantly higher in patients presenting with preoperative seizures compared with those without seizures (*p* < 0.001). In contrast, no significant difference in LDH levels was observed between male and female patients (*p* = 0.521). No significant association was observed between LDH levels and neuroanatomical tumor location (*p* = 0.115). No significant correlation was observed between serum LDH levels and patient age (Spearman *r* = − 0.002, *p* = 0.990). Similarly, no significant association was observed between serum LDH levels and extracranial metastatic disease (*p* = 0.322). Exploratory analyses comparing serum LDH levels and PTBE presence between previously untreated and previously treated patients revealed no significant differences in serum LDH levels (*p* = 0.804) or PTBE presence (*p* = 0.558), suggesting that prior systemic treatment status did not substantially confound the observed associations in the present cohort. No significant difference in serum LDH levels was observed between patients with and without prior radiotherapy (*p* = 0.132). Additionally, patients who received preoperative corticosteroids (*n* = 30) demonstrated significantly higher serum LDH levels compared to those who did not (*p* < 0.001). Spearman correlation analyses revealed no significant association between serum LDH and CRP (*r* = − 0.043, *p* = 0.792) or leukocyte count (*r* = 0.115, *p* = 0.472), supporting the interpretation that elevated LDH reflects tumor-specific biological activity rather than non-specific systemic inflammation. Imaging evidence of intratumoral necrosis was present in all 56 patients (100.0%), precluding comparative analysis. Furthermore, no significant difference in serum LDH levels was observed between patients with and without lymph node metastases (*p* = 0.682). Overall, these exploratory findings suggest an association between serum LDH and both tumor burden and proliferative activity, as well as clinically relevant manifestations such as preoperative seizures. These observations are consistent with LDH serving as a potential surrogate of tumor-related biological activity, though independent effects cannot be established from the present data.

### Discriminative performance of LDH, MIB-1 and tumor volume for preoperative PTBE

**Fig. 4:**
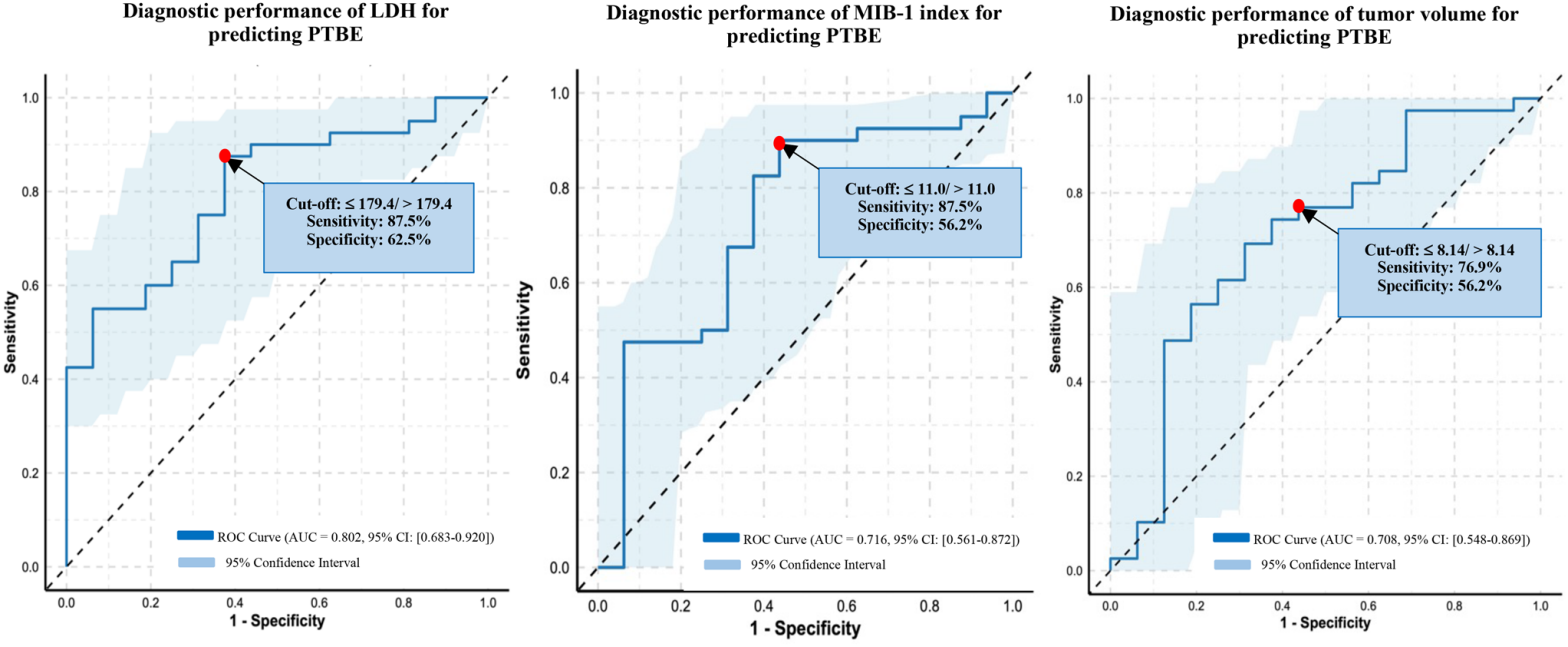
Receiver operating characteristic (ROC) curves illustrating the discriminative ability of serum LDH, MIB-1 proliferation index and tumor volume as surrogate biomarkers of PTBE. These analyses are not intended to replace MRI-based PTBE assessment, but provide a standardized metric for the strength of association between each biomarker and PTBE presence. (**A**) Serum LDH, (**B**) MIB-1 index, and (**C**) tumor volume. ROC curves demonstrate the discriminative performance of each variable for predicting the presence of preoperative PTBE. Serum LDH showed the highest discriminative ability (AUC = 0.802, 95% CI: 0.683–0.920, *p* < 0.001), followed by the MIB-1 index (AUC = 0.716, 95% CI: 0.561–0.872, *p* = 0.012) and tumor volume (AUC = 0.708, 95% CI: 0.548–0.869, *p* = 0.016). The red dots indicate the optimal cut-off values determined by the Youden index, with corresponding sensitivity and specificity displayed in each panel

To quantify the discriminative ability of serum LDH levels, MIB-1 index and tumor volume as surrogate biomarkers of edema burden receiver operating characteristics (ROC) curve analyses were performed, with corresponding ROC curves illustrated in Fig. 4. These analyses were not designed to replace MRI-based PTBE assessment, but rather to provide a standardized metric for the strength of the association between each biomarker and the presence of PTBE. Serum LDH showed the highest discriminative performance, with an area under the curve (AUC) of 0.802 (95% CI: 0.683–0.920, *p* < 0.001). Based on the Youden index, the optimal cut-off value for LDH was 179.4 U/L, corresponding to a sensitivity of 87.5% and specificity of 62.5% for predicting PTBE. The MIB-1 proliferation index also showed an association with PTBE with an AUC of 0.716 (95% CI: 0.561–0.872, *p* = 0.012). The optimal cut-off value determined by the Youden index was 11%, yielding a sensitivity of 87.5% and specificity of 56.2%. Similarly, tumor volume demonstrated significant predictive performance with an AUC of 0.708 (95% CI: 0.548–0.869, *p* = 0.016). The optimal cut-off was 8.14 cm^3^, corresponding to a sensitivity of 76.9% and specificity of 56.2%. Overall, serum LDH demonstrated the highest discriminative performance among the analyzed variables. However, exploratory pairwise comparisons using DeLong´s test revealed no statistically significant differences between the AUCs of LDH, MIB-1 and tumor volume (LDH vs. MIB-1: Z = 1.00, *p* = 0.319; LDH vs. tumor volume: Z = 1.17, *p* = 0.242; MIB-1 vs. tumor volume: Z = 0.09, *p* = 0.930).

## Discussion

In the present study, we investigated clinical and biological factors associated with the development of PTBE in patients with melanoma BM. We found that elevated serum LDH levels were significantly associated with the presence of PTBE and strongly correlated with the extent of edema. In addition, LDH levels were associated with tumor proliferative activity, tumor burden and the occurrence of preoperative seizures. Among the analyzed variables, LDH demonstrated the highest discriminative performance for predicting PTBE in ROC analysis. It should be noted that these analyses were not intended to position LDH as a clinical alternative to MRI-based PTBE assessment, but rather to provide a quantitative measure of the strength of the association between LDH and PTBE, complementing rather than replacing preoperative neuroimaging. Taken together, these exploratory findings suggest that serum LDH may be associated with tumor-related biological activity associated with the presence and severity of PTBE. Importantly, the observed associations between LDH and PTBE may at least partially reflect the relationship between LDH, tumor burden and proliferative tumor activity. Of note, the significant correlation between LDH and tumor volume observed in the present cohort raises the possibility that the association between LDH and PTBE is at least partially mediated by tumor burden, given that larger tumor volumes are independently associated with more extensive peritumoral edema. Furthermore, serum LDH elevation in solid tumors reflects not only tumor volume but also proliferative activity [21]. This is supported by the significant correlation between serum LDH and MIB-1 observed in the present cohort, consistent with Dong et al.^21^, who demonstrated that tumor LDH-A expression correlates significantly with MIB-1 proliferative activity. Notably, MIB-1 emerged as the only independent predictor of PTBE in the multivariable analysis, suggesting that the proliferative component of LDH elevation may represent the more direct biological driver of edema formation. In an exploratory multivariable logistic regression, serum LDH did not retain independent statistical significance, while MIB-1 proliferation index and preoperative seizure occurrence remained significant. This finding supports the interpretation that the association between LDH and PTBE may be mediated through tumor proliferative activity rather than reflecting an independent biological effect and reinforces the exploratory nature of all presented analyses. In this context, PTBE may represent one of several downstream correlates of biologically aggressive and metabolically active disease, though the present design does not permit conclusions regarding the directionality or independence of this relationship.

These findings extend and refine previous observations regarding the role of LDH in melanoma BM. Elevated LDH levels are a well-established prognostic marker in metastatic melanoma and are incorporated into staging systems such as the AJCC TNM classification [22]. Several studies have demonstrated that increased LDH levels are associated with poorer survival, a higher risk of BM and a more aggressive disease course [14, 15]. Notably, a retrospective analysis demonstrated that LDH levels exceeding twice the upper limit of normal identified patients with poor prognosis who were unlikely to benefit from encephalic radiotherapy [23]. Furthermore, serum LDH has been independently identified as a promising prognostic marker in patients with brain metastases across multiple tumor entities, further supporting its role as a clinically applicable biomarker for preoperative risk assessment [24]. However, direct associations between LDH and local radiological characteristics, such as PTBE, remain scarce and have largely been limited to qualitative assessment. To our knowledge, the present study is the first to demonstrate a strong quantitative association between preoperative serum LDH levels and volumetrically measured PTBE in melanoma BM.

The consistent associations between LDH, edema volume, tumor proliferation, tumor burden and seizure occurrence suggest that LDH may reflect both biologically aggressive disease and clinically relevant neurological manifestations. In particular, the correlation between LDH and MIB-1 index is consistent with a link to tumor proliferative activity, while the association with tumor volume may reflect tumor burden. These findings are in line with previous studies demonstrating that elevated LDH levels are associated with increased tumor growth, higher proliferative capacity and more advanced disease stages in metastatic melanoma [15, 22, 25]. Furthermore, the observed associations between elevated LDH levels and preoperative seizures highlight the clinical relevance of this biomarker, suggesting that LDH is associated not only to radiological features but also to symptomatic disease presentation. In this context, increased tumor burden and PTBE have been shown to contribute to cortical irritation and seizure development in patients with BM [26, 27]. The pathophysiological link between peritumoral edema and seizure development is well established, with PTBE contributing to cortical irritation and neuronal hyperexcitability through mechanisms including focal ischemia, ion channel disruption and mechanical cortical compression [28]. Taken together, these exploratory findings are consistent with the hypothesis that LDH may serve as an integrative surrogate of tumor biology, tumor burden and neurological symptomatology, though causality cannot be inferred from the present data. However, the present findings should be interpreted with caution, as LDH is unlikely to represent a direct mediator of edema formation. Exploratory analyses did not demonstrate a significant association between extracranial metastatic disease and either serum LDH levels or PTBE occurrence in the present cohort. Although these findings do not exclude a contribution of systemic tumor burden, they suggest that the observed relationship between LDH and PTBE may not be solely attributable to extracranial metastatic disease. Furthermore, exploratory analyses comparing serum LDH levels and PTBE presence between previously untreated and previously treated patients revealed no significant differences, suggesting that prior systemic treatment status did not substantially confound the observed associations in the present cohort. Whether targeted therapies such as BRAF/ MEK inhibitors or immune checkpoint inhibitors specifically modulate the relationship between serum LDH and PTBE formation represents an interesting avenue for future investigation. BRAF/ MEK inhibition has been shown to reduce serum LDH as a dynamic marker of treatment response in metastatic melanoma and elevated baseline LDH has been identified as a negative predictive factor for response to targeted therapy [29]. Disentangling the independent contributions of these therapies to both LDH levels and PTBE formation would require larger cohorts with detailed longitudinal treatment characterization and represents a key objective for future prospective studies.

From a pathophysiological perspective, this relationship appears biologically plausible. LDH is considered a marker of increased tumor cell turnover, hypoxia, and necrosis in aggressive metastatic malignancies [30]. Hypoxia-related signaling pathways have been proposed to promote the release of pro-inflammatory cytokines and angiogenic factors, particularly vascular endothelial growth factor (VEGF), which are associated with increased vascular permeability and disruption of the BBB processes that have been implicated in the development of vasogenic PTBE. However, it is equally plausible that the directionality of this relationship is reversed: larger, more necrotic and more edematous tumors may release greater amounts of LDH into the circulation, such that the elevated LDH may present a consequence of, rather than a correlated contributing to, PTBE. The present cross-sectional study design does not permit conclusions regarding the directionality of this association and the observed correlation between LDH and PTBE volume should be interpreted as a statistical association rather than evidence of a mechanistic relationship.

It should further be noted that the relationship between PTBE and tumor biological aggressiveness is not uniform across all brain metastasis entities and that greater edema volume does not necessarily indicate a more aggressive tumor phenotype. Recent data in lung adenocarcinoma brain metastases demonstrated that TTF-1-negative tumors associated with significantly worse survival and higher rates of intracranial progression did not exhibit correspondingly greater PTBE despite their more aggressive clinical course [31]. Similarly, Meyer et al. found no significant linear associations between perifocal edema volume and immunohistochemical markers of proliferative potential, microvessel density, neoangiogenesis or invasiveness in a mixed brain metastasis cohort [32]. Furthermore, Kerschbaumer et al. demonstrated that the relationship between edema volume and survival in patients with cerebral metastases is complex and does not follow a straightforward linear pattern [33]. Collectively, these findings underscore that PTBE should not be interpreted as a universal surrogate of tumor aggressiveness across metastatic entities. The associations observed in the present melanoma cohort may therefore reflect melanoma-specific biological interactions and should not be generalized beyond this disease context.

These findings may have clinically relevant implications and warrant further investigation. Preoperative serum LDH is a routinely available laboratory parameter as was associated with the presence of PTBE in the present cohort [22, 25, 26]. If confirmed in larger prospective studies, such associations could hypothetically inform preoperative risk assessment and heightened clinical vigilance for neurological complications such as seizures or focal deficits. Such heightened vigilance is particularly relevant given current guidelines recommending individualized antiepileptic management in patients with BM based on individual clinical risk factors, including prior seizure history and the presence of cortical edema [34]. This is particularly relevant in light of recent clinical evidence demonstrating that cumulative perioperative dexamethasone exposure is independently associated with worse overall survival in patients undergoing brain metastasis resection, with doses ≥ 122 mg linked to significantly poorer outcomes in a larger multicenter cohort of 1,064 patients [35]. Moreover, the combined use of corticosteroids with immune checkpoint inhibitors has been associated with a significant mortality disadvantage in brain metastasis patients, as demonstrated in a systematic review and meta-analysis [36]. These data collectively highlight the clinical importance of minimizing unnecessary corticosteroid exposure, particularly in patients receiving immunotherapy and underscore the potential value of preoperatively identifying patients at elevated risk for PTBE. However, given the retrospective design, small sample size and exploratory nature of the analyses, no direct recommendations for individualized perioperative management including specific adjustments to dexamethasone therapy can be derived from the present data. In particular, patients presenting with overt seizures and PTBE on preoperative imaging would already receive antiepileptic and corticosteroid therapy as standard of care, independent of serum LDH levels [35, 36]. The potential added value of serum LDH may lie in its role as a routinely available preoperative laboratory parameter that could hypothetically contribute to risk awareness in patients without overt clinical signs of PTBE, or as one component of a future multiparameter risk stratification model, pending prospective validation. Furthermore, LDH is already integrated into established prognostic models such as the melanoma-specific GPA index [37, 38]. The present findings extended the descriptive associations of LDH by linking it to a local radiological characteristic of BM. However, whether this translates into improved prediction of neurological outcomes requires prospective validation. Elevated serum LDH reflects tumor-derived lactate accumulation, which promotes an immunosuppressive microenvironment and has been associated with reduced response to immune checkpoint inhibitors in metastatic melanoma [14, 39–41]. Whether serum LDH carries predictive value for intracranial immunotherapy response in this setting remains speculative and warrants dedicated prospective investigation.

The present study has several limitations. First, the retrospective design and relatively small patient cohort may introduce selection bias and limit the statistical power of the analysis. Furthermore, the extended study period spanning 12 years (2012–2024) may have introduced temporal heterogeneity, as systemic treatment protocols, perioperative management strategies and imaging standards evolved considerably during this time, representing an additional source of potential bias. Second, the single-center nature of the study may restrict the generalizability of the findings. In addition, the relatively low prevalence of BRAF-mutated tumors in the present cohort may reflect selection bias inherent to the surgically treated patient population.

Furthermore, segmentation was performed by a single rater without formal interrater reliability assessment, which may introduce a degree of measurement variability despite the standardized workflow. Additionally, PTBE was assessed using FLAIR sequences where available and T2-weighted sequences otherwise. As T2 does not suppress CSF signal, edema volumes may have been overestimated in cases assessed on T2, representing a potential source of measurement variability. Although extracranial metastatic disease was exploratively analyzed, overall systemic tumor burden was not quantitatively characterized and may therefore still represent an important confounding factor influencing serum LDH levels. Extracranial disease burden was assessed only as a binary variable, without quantification of metastatic load across organ systems. As LDH is an established marker of systemic tumor activity, a more granular characterization of extracranial burden would have been necessary to disentangle intracranial from systemic contributions to LDH elevation. As LDH is an established systemic biomarker in metastatic melanoma, elevated LDH levels may at least partially reflect overall systemic tumor activity rather than exclusively intracranial disease characteristics and may additionally be influenced by various non-tumor related systemic conditions [42, 43]. Moreover, longitudinal perioperative LDH measurements were not available, limiting the assessment of dynamic biomarker changes following tumor resection. Exploratory analyses evaluating associations between preoperative LDH and postoperative clinical outcomes including overall survival, functional status assessed by the KPS, surgical complications and postoperative seizure occurrence did not yield statistically significant results in the present cohort, likely reflecting the limited sample size, high censoring rate and selection bias inherent to a surgically treated patient population. Demonstrating a postoperative decline in serum LDH levels concurrent with edema resolution would provide important additional support for the proposed association and represents a key objective for future prospective studies. Finally, the quantification of edema volume was based on radiological segmentation, which, despite standardized methods, may be subject to a certain degree of measurement variability.

## Conclusion

In summary, the findings of this study demonstrate that elevated serum LDH levels are closely associated with both the presence and extent of PTBE in patients with melanoma BM. In addition, LDH levels were associated with tumor proliferative activity, tumor burden and clinically relevant manifestations such as preoperative seizures, suggesting that LDH may serve as a surrogate marker of biologically aggressive tumor activity associated with PTBE formation. These exploratory findings highlight LDH as a routinely available laboratory parameter that warrants further investigation as a preoperative risk stratification biomarker. Prospective validation in larger, ideally multicenter cohorts is necessary before any implications for individualized perioperative management can be drawn. Further prospective studies incorporating quantitative assessment of systemic tumor burden and longitudinal perioperative LDH measurements including postoperative follow-up to assess dynamic biomarker changes concurrent with edema resolution and systemic documentation of clinical outcomes, including response to immunotherapy and stereotactic radiosurgery, local recurrence rates and long-term functional status, are warranted to validate these findings and further define the clinical role of LDH in patients with melanoma BM.

## Data Availability

No datasets were generated or analysed during the current study.
